# Overexpression of HIF-2****α****, TWIST, and CXCR4 Is Associated with Lymph Node Metastasis in Papillary Thyroid Carcinoma

**DOI:** 10.1155/2013/589423

**Published:** 2013-10-29

**Authors:** Ni Wang, Hao-Jun Luo, Guo-Bing Yin, Chao-Ran Dong, Man Xu, George G. Chen, Zhi-Min Liu

**Affiliations:** ^1^Department of Biochemistry and Molecular Biology, Molecular Medicine and Cancer Research Center, Chongqing Medical University, 1 Yixueyuan Road, Yuzhong District, Chongqing 400016, China; ^2^Department of Breast and Thyroid Surgery, The Second Affiliated Hospital, Chongqing Medical University, Chongqing 400016, China; ^3^Department of Pathology, Molecular Medicine and Cancer Research Center, Chongqing Medical University, Chongqing 400016, China; ^4^Department of Surgery, The Chinese University of Hong Kong, Prince of Wales Hospital, Shatin, N.T., Hong Kong

## Abstract

This study aimed to examine HIF-2**α**, TWIST, and CXCR4 expression in papillary thyroid carcinoma (PTC) and assesses the association of their expression with clinicopathological indicators. HIF-2**α**, TWIST, and CXCR4 protein expression in 129 PTCs, 61 nodular hyperplasia, and 118 normal thyroid tissue specimens was analyzed using immunohistochemistry. The protein expression levels of these three molecules were upregulated in PTCs. High protein expression of HIF-2**α**, TWIST, and CXCR4 was significantly correlated with lymph node metastasis (LNM) (*P* < 0.001). Furthermore, HIF-2**α**, TWIST, and CXCR4 protein expression was correlated with one another. Concomitant high expression of these molecules had stronger correlation with LNM than did each alone (*P* = 0.032 for HIF-2**α**/TWIST, *P* < 0.001 for HIF-2**α**/CXCR4, *P* = 0.018 for TWIST/CXCR4, and *P* < 0.001 for HIF-2**α**/TWIST/CXCR4). Additionally, HIF-2**α**, TWIST, and CXCR4 mRNA expression were assessed in 30 PTCs, 10 nodular hyperplasia, and 10 normal thyroid tissue specimens using real-time RT-PCR. TWIST and CXCR4 mRNA expression levels were up-regulated in PTCs, and high mRNA expression of TWIST and CXCR4 was significantly correlated with LNM (*P* = 0.005 and *P* = 0.010, resp.). These results demonstrated that the evaluation of HIF-2**α**, TWIST, and CXCR4 expression in PTC may be useful in predicting the risk of LNM.

## 1. Introduction

Papillary thyroid carcinoma (PTC) accounts for 80% of thyroid malignancy and is characterized by slow growth and an excellent prognosis. However, some cases show relatively early recurrence, severe invasion, lymph node metastasis (LNM), or distant metastasis [1]. It is important to identify the characteristics of PTC that have a high risk for invasion and metastasis.

Intratumoral hypoxia is an independent indicator of poor prognosis and contributes to a more aggressive tumor phenotype [2]. Tumor adaptation to hypoxia is predominantly regulated by two structurally related hypoxia inducible factors (HIFs), HIF-1*α* and HIF-2*α* [3, 4]. They activate the expression of genes involved in tumor growth, metabolism, angiogenesis, and metastasis. Although highly homologous, HIF-1*α* and HIF-2*α* play critical but nonoverlapping roles in tumor progression [5, 6]. Recent studies have shown that TWIST and CXCR4 are two direct target genes of HIF-2*α*, HIF-2*α* can activate the expression of TWIST and CXCR4, which subsequently promote invasion and metastasis of tumor cells [7–9].

TWIST is a class II member of basic helix-loop-helix (bHLH) transcription factors and is thought to regulate epithelial-mesenchymal transition (EMT) through the downregulation of key proteins that maintain epithelial cell characteristics and upregulation of proteins that confer a mesenchymal phenotype [10]. TWIST is overexpressed in many types of tumors including breast, gastric, hepatocellular, prostate, and bladder cancers. Its upregulation correlates with high cancer aggressiveness and poor patient survival rate [11, 12]. However, at present, studies barely assess the expression of TWIST and its role in PTC.

Chemokine receptors are transmembrane proteins that interact with specific chemokine ligands, resulting in G-protein-coupled signal transduction leading to chemotaxis or directional movement along a chemical gradient. Recently, functional chemokine receptors have shown to be expressed by a large number of human malignancies, leading to the hypothesis that chemokines may stimulate proliferation, chemotaxis, and site-directed metastasis of tumor cells [13, 14]. The CXC chemokine receptor 4 (CXCR4) interacts specifically with its chemokine ligand, stromal cell-derived factor 1 (SDF-1/CXCL12), to exert proliferative and chemotactic effects in CXCR4-expressing cancer cells, accounting for an association of CXCR4 with metastasis in numerous human tumors, including lung, breast, prostate, colon, and thyroid cancer [15–19]. 

Given their association with tumor invasion and metastasis, the purpose of this study was to examine HIF-2*α*, TWIST, and CXCR4 expression in PTC, to assess association of HIF-2*α*, TWIST, and CXCR4 expression with several clinicopathological indicators, and to evaluate potential usefulness of the three molecules in prediction for invasion and metastasis of PTC.

## 2. Materials and Methods

### 2.1. Case Selection and Tissue Sample Preparation

Tumor specimens for immunohistochemistry were obtained from 129 PTC patients who underwent thyroidectomy in the Department of Surgery, the First Affiliated Hospital, Chongqing Medical University, China between January 2010 and January 2013. There were 32 men and 97 women with a median age of 45 years. According to histopathologic diagnosis, there were 80 classic PTC, 21 follicular variant of PTC, 15 tall cell variant of PTC, and 13 oncocytic variant of PTC. The size of primary tumor ranged from 0.3 to 6.0 cm (2.28 ± 1.38). According to AJCC classification [20], there were 69 patients with stage I and stage II 60 with stage III and stage IV. Sixty-eight patients were confirmed to have LNM. Besides, benign thyroid disease specimens were obtained from 61 patients with nodular hyperplasia. One hundred and eighteen normal thyroid tissues were taken from the contralateral lobe of PTC specimens, which exhibit apparently normal morphology as a control. The study protocol was approved by the Research Ethics Committee of Chongqing Medical University and informed consent was obtained from all patients. 

Tumor specimens for real-time RT-PCR were obtained from 30 PTC patients between January 2012 and January 2013, including 15 PTCs without LNM and 15 PTCs with LNM. The benign thyroid disease specimens were obtained from 10 patients with nodular hyperplasia. For controls, 10 normal thyroid tissue specimens were used. All specimens were immediately snap-frozen in liquid nitrogen and stored at −80°C up to subsequent real-time RT-PCR.

### 2.2. Tissue Microarray

Formalin-fixed, paraffin-embedded blocks were routinely prepared from surgical specimens of PTC, nodular hyperplasia, and normal thyroid tissue. Representative areas containing tumor, nodular hyperplasia, or normal thyroid tissue were identified by a pathologist. Duplicate tissue cores with a diameter of 0.6 mm were taken from each specimen (Beecher Instruments, Silver Springs, USA) and arrayed on a recipient paraffin block, using standard procedures [21]. Serial 5-*μ*m-thick sections were cut with a Leica microtome (Leica Microsystems; Wetzlar, Germany) and mounted onto polylysine-coated slides.

### 2.3. Immunohistochemical Staining

Sections from TMA blocks were dewaxed and hydrated. Antigen retrieval was achieved by microwaving in 0.01 M citrate buffer (pH 6.0) for 10 min. After microwave treatment, the slides were treated with 3% hydrogen peroxide for 30 min to block the endogenous peroxidase and followed by blocking with 10% normal goat serum (50062Z, Invitrogen, USA) in PBS at room temperature for 1 h. The slides were then incubated overnight at 4°C in the primary rabbit polyclonal anti-HIF-2*α* antibody (1 : 50 dilution, ab20654; Abcam, USA), anti-TWIST antibody (1 : 50 dilution, ab49254; Abcam, USA), or anti-CXCR4 antibody (1 : 50 dilution, ab7199; Abcam, USA). For negative isotype controls, the sections were incubated in rabbit immunoglobulin G (1 : 1000, NI01-100UG; Merck Millipore, Germany). After defrosting at 37°C for 30 min, the slides were washed with PBS and incubated with a secondary biotinylated goat-anti-rabbit antibody (ZB-2010; Zhongshan Golden Bridge Biotechnology, China) for 30 min, peroxidase-labeled streptavidin (ZB-2404; Zhongshan Golden Bridge Biotechnology, China) for 20 min, and diaminobenzidine chromogen substrate (Sigma, USA) for 5 min. Slides were counterstained with hematoxylin, dehydrated, and mounted. 

### 2.4. Immunohistochemical Scoring

A semiquantitative assessment of immunohistochemical (IHC) scoring was performed by two observers blinded to the diagnosis. The IHC score was assigned based on staining intensity and percentage of positive cells. The intensity score was assigned as 0 (no staining), 1 (weak staining), 2 (moderate staining), and 3 (strong staining). The proportion score was assigned as 0 (<5% positive cells), 1 (6–25% positive cells), 2 (26–50% positive cells), 3 (51–75% positive cells), and 4 (>75% positive cells). Multiplication of the intensity and percentage scores gave rise to the final staining score: 0 (negative), + (1–4), ++ (5–8), and +++ (9–12). For statistical analysis, a final staining score of negative or + was combined into the low expression group, and a final staining score of ++ or +++ was combined into the high expression group.

### 2.5. RNA Extraction, Reverse Transcription, and Real-Time PCR

Total RNA was extracted from frozen thyroid tissues using TRIzol reagent (Invitrogen, Camarillo, CA, USA), and residual genomic DNA was eliminated by DNase I digestion (Ambion, USA). RNA purity was confirmed by spectrophotometry. Total RNA was reverse transcribed to cDNA by using SuperScript III Reverse Transcriptase (Invitrogen, USA) according to the manufacturer's protocol. The final cDNA product was amounted to 25 *μ*L and stored at −80°C.

Real-time PCR was performed by using SYBR-Green real-time PCR method on the ABI-Prism 7000 sequence detector (Applied Biosystems, USA). The primers are shown in Table 1. The predicated product size of the primers for HIF-2*α*, TWIST, and CXCR4 was 196 bp, 207 bp, and 109 bp, respectively. Quantities of gene specific mRNA expression were determined by the CT method. Samples were analyzed in triplicate. Average threshold cycle (CT) values for glyceraldehyde-3-phosphate dehydrogenase (GAPDH) were used as an internal calibrator. The 2^−ΔΔCT^ method was used for relative quantitation [22]. Results are presented as the mean ± standard deviation of three independent experiments. The real-time PCR mix was made on the basis of the prescription from the supplier: 6 *μ*L sterile water, 1 *μ*L sense and 1 *μ*L antisense primers, 10 *μ*L Platinum SYBR Green qPCR SuperMix-UDG w/ROX (Invitrogen, USA), and 2 *μ*L target cDNA in a total volume of 20 *μ*L. Run conditions were 50°C for 2 min, 95°C for 10 min, followed by 40 cycles at 95°C for 15 s and 60°C for 1 min.

### 2.6. Statistical Analysis

Statistical analysis was performed using SPSS 18.0 statistical software. Data are presented as percentages and mean and standard deviation, according to the distribution. Significance was assessed using chi-square, Spearman rank, and Mann-Whitney *U* tests as appropriate, to compare the groups. *P* value <0.05 was considered statistically significant.

## 3. Results

### 3.1. Immunohistochemical Expression of HIF-2*α*, TWIST, and CXCR4 in PTCs, Nodular Hyperplasia, and Normal Thyroid Tissues

**Figure 1:**
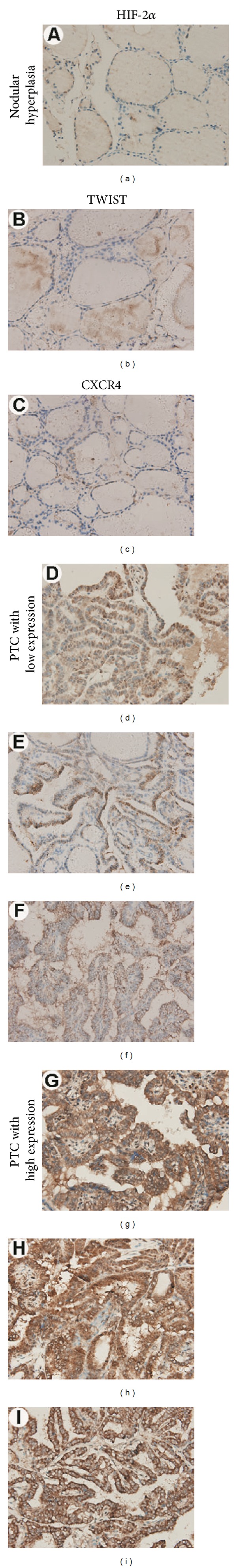
Immunohistochemical staining for HIF-2*α*, TWIST, and CXCR4. Columns correspond to immunostaining for HIF-2*α*, TWIST, and CXCR4, respectively. The first row exhibits weak staining of nodular hyperplasia tissues with the indicated antibody ((a)–(c)); the succeeding rows show moderate staining ((d)–(f)) and strong staining ((g)–(i)) of HIF-2*α*, TWIST, and CXCR4 in PTCs. All the pictures are in high-power fields (×400).

HIF-2*α*, TWIST, and CXCR4 protein expression was examined by immunohistochemistry and illustrated in Figure 1. The immunoreactivities of HIF-2*α* and TWIST were detected in the cytoplasm and nuclei and CXCR4 in the cytoplasm and cell membrane. In nodular hyperplasia tissues, there were only a few follicular cells with weak staining for HIF-2*α* (a), TWIST (b), and CXCR4 (c). However, in PTCs, some cases had quite a few tumor cells with moderate staining for these three molecules (d)–(f), and some cases had a lot of tumor cells with strong staining for the three molecules (g)–(i). As shown in Tables 2 and 3, like the normal thyroid tissues, the majority of nodular hyperplasia tissues have negative or 1 IHC score; no cases showed high expression (≥5) of these three molecules. However, in PTCs, the majority of cases have ≥3 IHC score; high expression (≥5) was present in 64 (49.6%), 56 (43.4%), and 71 (55.0%) of 129 cases for HIF-2*α*, TWIST, and CXCR4, respectively. The differences in HIF-2*α*, TWIST, and CXCR4 protein expression levels between PTCs and normal thyroid tissues as well nodular hyperplasia tissues were statistically significant (*P* < 0.001). 

### 3.2. Correlation of HIF-2*α*, TWIST, and CXCR4 Protein Expression with Clinicopathological Features in PTCs

The correlation of HIF-2*α*, TWIST, and CXCR4 protein expression with clinicopathological data was assessed by chi-square test and summarized in Table 3. There were no significant differences in HIF-2*α*, TWIST, and CXCR4 protein expression between patients with different histologic subtypes of PTC (*P* = 0.980, *P* = 0.963, and *P* = 0.672, resp.), between older (>45) and younger (≤45) patients (*P* = 0.935, *P* = 0.159, and *P* = 0.483, resp.), between male and female patients (*P* = 0.960, *P* = 0.437, and *P* = 0.570, resp.), between patients with larger (>2.3) and smaller (≤2.3) tumor size (*P* = 0.243, *P* = 0.135, and *P* = 0.322, resp.), and between patients with higher (III-IV) and lower stage (I-II) (*P* = 0.935, *P* = 0.487, and *P* = 0.993, resp.). However, HIF-2*α*, TWIST, and CXCR protein expression was significantly correlated with LNM; PTC patients with LNM showed higher protein expression of these three molecules than those without LNM (*P* < 0.001 for all the three molecules).

### 3.3. Correlation of HIF-2*α*, TWIST, and CXCR4 Protein Expression with One Another in PTCs

The correlation of HIF-2*α*, TWIST, and CXCR4 protein expression with one another was assessed by Spearman rank test. As shown in Table 4, 40/129 PTCs showed high expression and 49/129 displayed low expression for both HIF-2*α* and TWIST. The correlation between HIF-2*α* and TWIST expression was statistically significant (*r*_*s*_ = 0.382, *P* < 0.001). Similarly, there was a statistically significant correlation between expression of HIF-2*α* and CXCR4 (*r*_*s*_ = 0.398, *P* < 0.001). For both HIF-2*α* and CXCR4, 48/129 PTCs showed high expression. In addition, high expression for both TWIST and CXCR4 was present in 44/129 PTCs. A significantly positive correlation (*r*_*s*_ = 0.414, *P* < 0.001) was also present between expression of TWIST and CXCR4.

### 3.4. Association of Concomitant HIF-2*α*, TWIST, and CXCR4 High Expression with LNM in PTCs

**Figure 2:**
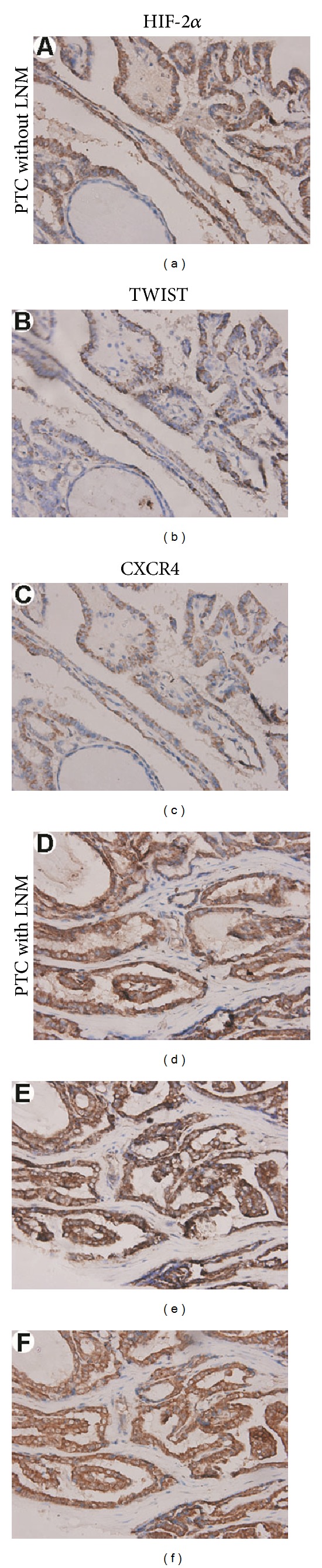
Association of concomitant HIF-2*α*, TWIST, and CXCR4 high expression with LNM in PTCs. Columns correspond to immunostaining for HIF-2*α*, TWIST, and CXCR4, respectively. The first row is the immunostaining of a representative of PTC without LNM showing only HIF-2*α* high expression (a), low expression of TWIST (b), and CXCR4 (c); the second row is the immunostaining of a representative of PTC with LNM showing high expression of HIF-2*α* (d), TWIST (e), and CXCR4 (f). All the pictures are in high-power fields (×400).

Given that HIF-2*α*, TWIST, and CXCR4 protein expression were correlated with one another and statistical analysis showed that the incidence of LNM tends to be higher in PTCs with high protein expression of HIF-2*α*, TWIST, and CXCR4, we further evaluated the correlation of LNM with concomitant high expression of HIF-2*α*/TWIST, HIF-2*α*/CXCR4, or TWIST/CXCR4. As shown in Table 5, the incidence of LNM is significantly higher in patients (87.5%) with high expression of HIF-2*α*/TWIST than in those patients (67.5%) with high expression of only one of these two molecules or in those patients (12.2%) without high expression for either of these two molecules. Similar results were observed in PTCs with high expression of HIF-2*α*/CXCR4 and TWIST/CXCR4. There were statistically significant differences in the incidence of LNM between patients with high expression of only one and any two of the three molecules (*P* = 0.032 for HIF-2*α*/TWIST, *P* < 0.001 for HIF-2*α*/CXCR4, and *P* = 0.018 for TWIST/CXCR4). In addition, statistical analysis showed that concomitant high expression of all the three molecules is significantly associated with LNM as compared with cases not showing such expression (*P* < 0.001). As demonstrated in Figures 2(a)–2(c) are representative of PTC without LNM showing only HIF-2*α* high expression and low expression of the other two molecules, TWIST and CXCR4; (d)-(e) are representative of PTC with LNM showing high expression of all the three molecules, HIF-2*α*, TWIST, and CXCR4, respectively.

### 3.5. Correlation of HIF-2*α*, TWIST, and CXCR4 mRNA Expression with LNM in PTCs

To compare gene expression of HIF-2*α*, TWIST, and CXCR4 in PTCs without and with LNM, fifteen PTCs without LNM and another fifteen PTCs with LNM were collected to analyze HIF-2*α*, TWIST, and CXCR4 mRNA levels using real-time RT-PCR. Ten nodular hyperplasia and ten normal thyroid tissues were used for comparison and as a control. As shown in Table 6, mean HIF-2*α* mRNA levels were 2.9 ± 0.6-fold in normal thyroid tissues, 3.1 ± 1.3-fold in nodular hyperplasia tissues, 3.7 ± 1.3-fold in PTCs without LNM, and 4.3 ± 1.7-fold in PTCs with LNM. There was no statistically significant difference in HIF-2*α* mRNA levels between nodular hyperplasia and normal thyroid tissues (*P* = 0.940), between PTCs and normal thyroid tissues (*P* = 0.101), and between PTCs with and without LNM (*P* = 0.467). In contrast, TWIST and CXCR4 mRNA levels were significantly higher in PTCs compared with normal thyroid tissues (*P* < 0.001 for both), while there were no statistically significant differences in TWIST and CXCR4 mRNA levels between nodular hyperplasia and normal thyroid tissues (*P* = 0.111 and *P* = 0.226, resp.). Moreover, PTCs with LNM showed increased mRNA levels of TWIST and CXCR4 compared with those without LNM. The differences in TWIST and CXCR4 mRNA levels between PTCs with and without LNM were statistically significant (*P* = 0.005 and *P* = 0.010, resp.). 

## 4. Discussion

Hypoxia is a common condition found in a wide range of solid tumors and has been increasingly recognized to play a central role in different stages of tumor progression [2]. Tumor adaptation to hypoxia is predominantly regulated by HIF-1*α* and HIF-2*α*, which activate the expression of genes involved in proliferation, metabolism, angiogenesis, and metastasis. While highly homologous, HIF-1*α* and HIF-2*α* have unique tissue distributions and play critical but nonoverlapping roles in tumor progression [5, 6]. HIF-2*α* promotes metastasis through regulation of critical factors controlling tumor cell metastatic potential, such as CXCR4 and TWIST [7–9]. To date, studies have shown that HIF-2*α*, TWIST, and CXCR4 are overexpressed in several human tumors such as clear cell renal carcinoma (ccRCC) [23], nonsmall cell lung cancer (NSCLC) [24], neuroblastoma [25], and breast, prostate, gastric, hepatocellular, colon, and bladder cancers [11–18]. CXCR4 has been observed to be upregulated in thyroid cancer [19]. However, studies barely examined HIF-2*α* and TWIST expression in PTC; moreover, no study investigated simultaneously the expression of HIF-2*α*, TWIST, and CXCR4 and assessed correlation of their expression with clinicopathological features in PTC. In our present study, we examined HIF-2*α*, TWIST, and CXCR4 protein expression in PTCs, nodular hyperplasia and normal thyroid tissues using immunohistochemistry. The results demonstrated that no cases of normal thyroid tissue and nodular hyperplasia show high protein expression of HIF-2*α*, TWIST, and CXCR4. However, in PTCs, high protein expression was present in 49.6%, 43.4%, and 55.0% of cases for HIF-2*α*, TWIST and CXCR4, respectively. The differences in HIF-2*α*, TWIST, and CXCR4 protein expression between PTCs and normal thyroid tissues as well nodular hyperplasia were statistically significant (*P* < 0.001). Then we assessed the correlation of HIF-2*α*, TWIST, and CXCR4 protein expression with several clinicopathological indicators. We found that HIF-2*α*, TWIST, and CXCR4 protein expression were not associated with histologic subtype, gender, age, tumor size, and TNM stage. However, there was a significant correlation between LNM and single HIF-2*α*, TWIST, and CXCR4 protein expression. High protein expression of HIF-2*α*, TWIST, and CXCR4 was associated with positive LNM. These results suggested that HIF-2*α*, TWIST, and CXCR4 may play an important role in invasion and metastasis of PTC. 

Additionally, our study, for the first time, demonstrated a significantly positive correlation between HIF-2*α*, TWIST, and CXCR4 expression in PTCs. HIF-2*α* expression is positively correlated with TWIST expression (*r*_*s*_ = 0.382, *P* < 0.001) and CXCR4 expression (*r*_*s*_ = 0.398, *P* < 0.001). This finding could be supported by several recent studies. Gort et al. reported that TWIST expression in human cancer cells is enhanced by hypoxia in a HIF-2*α*-dependent manner. TWIST is a direct target of HIF-2*α*, and HIF-2*α* can promote the invasion and migration of cancer cells by upregulation of TWIST [7]. Meanwhile, studies have also showed that HIF-2*α* can promote invasion and metastasis through upregulation of CXCR4 expression [8, 9]. In addition, a significantly positive correlation (*r*_*s*_ = 0.414, *P* < 0.001) was present between expression of TWIST and CXCR4. To date, there is no report to explain this positive correlation. It is necessary to further explore mechanisms underlying this correlation.

Given that HIF-2*α*, TWIST, and CXCR4 protein expression were positively correlated with one another and the expression of these single molecules was related to LNM, we subsequently evaluated the association of concomitant expression of HIF-2*α*, TWIST, and CXCR4 with LNM in PTCs. The results showed that concomitant expression of any two of these three molecules had stronger correlation with LNM than did each alone. Concomitant expression of all three molecules strongly correlates with LNM.

Lastly, we analyzed HIF-2*α*, TWIST, and CXCR4 mRNA expression levels in PTCs using real-time RT-PCR. The results demonstrated that TWIST and CXCR4 mRNA levels were significantly higher in PTCs than in normal thyroid tissues. Moreover, as their protein expression, TWIST and CXCR4 mRNA expression levels were also correlated with LNM; PTCs with LNM showed to have higher mRNA levels of TWIST and CXCR4 than those without LNM. However, there was no statistically significant difference in HIF-2*α* mRNA expression levels between PTCs, nodular hyperplasia, and normal thyroid tissues. Possibly, HIF-2*α*, like HIF-1*α* [3, 4], is predominantly regulated through hypoxia-dependent protein stabilization and genetic alterations, and under both normoxia and hypoxia, HIF-2*α* mRNA is also expressed constitutively in many cell types, including cancer cells.

## 5. Conclusions

In summary, our results, for the first time, demonstrated a positive correlation of HIF-2*α*, TWIST, and CXCR4 expression in PTCs. High expression of HIF-2*α*, TWIST, and CXCR4 was associated with LNM. Concomitant high expression of any two or all of the three molecules had stronger correlation with LNM than did each alone. Consequently, our results provide a possible basis for prediction of LNM in PTC. Future studies in larger sets of patients will be necessary to determine the utility of these molecules as biomarkers of tumor diagnosis and prognosis in PTC.

## Figures and Tables

**Table 1:** Primers used for real-time RT-PCR.

Gene	Primers	Product size
HIF-2*α*	Forward: 5′-TCTGAAAACGAGTCCGAAGCC-3′	196 bp
	Reverse: 5′-GGTCGCAGGGATGAGTGAAGT-3′	
TWIST	Forward: 5′-CGACGACAGCCTGAGCAACA-3′	207 bp
	Reverse: 5′-CCACAGCCCGCAGACTTCTT-3′	
CXCR4	Forward: 5′-CCACGCCACCAACAGTCAGA-3′	109 bp
	Reverse: 5′-GGCAAAGATGAAGTCGGGAATA-3′	
GAPDH	Forward: 5′-GGAGTCCACTGGCGTCTTCA-3′	191 bp
	Reverse: 5′-GGGGTGCTAAGCAGTTGGTG-3′	

**Table 2:** Immunohistochemical analysis of HIF-2*α*, TWIST, and CXCR4 expression in 129 PTCs, 61 nodular hyperplasia, and 118 normal thyroid tissue specimens according to the scoring system.

Score	HIF-2*α*			TWIST			CXCR4		
	Normal thyroid tissue *n* (%)	Nodular hyperplasia *n* (%)	PTC *n* (%)	Normal thyroid tissue *n* (%)	Nodular hyperplasia *n* (%)	PTC *n* (%)	Normal thyroid tissue *n* (%)	Nodular hyperplasia *n* (%)	PTC *n* (%)
0									
Negative	94 (79.7)	38 (62.3)	4 (3.1)	97 (82.2)	40 (65.6)	3 (2.3)	91 (77.1)	36 (59.0)	3 (2.3)
+									
1	21 (17.8)	18 (29.5)	7 (5.4)	19 (16.1)	16 (26.2)	12 (9.3)	23 (19.5)	19 (31.1)	8 (6.2)
2	3 (2.5)	4 (6.6)	16 (12.4)	2 (1.7)	5 (8.2)	17 (13.2)	4 (3.4)	4 (6.6)	13 (10.1)
3	0 (0)	1 (1.6)	18 (14.0)	0 (0)	0 (0)	19 (14.7)	0 (0)	2 (3.3)	16 (12.4)
4	0 (0)	0 (0)	20 (15.5)	0 (0)	0 (0)	22 (17.1)	0 (0)	0 (0)	18 (13.9)
++									
6	0 (0)	0 (0)	16 (12.4)	0 (0)	0 (0)	19 (14.7)	0 (0)	0 (0)	19 (14.7)
8	0 (0)	0 (0)	23 (17.8)	0 (0)	0 (0)	17 (13.2)	0 (0)	0 (0)	17 (13.2)
+++									
9	0 (0)	0 (0)	16 (12.4)	0 (0)	0 (0)	17 (13.2)	0 (0)	0 (0)	20 (15.5)
12	0 (0)	0 (0)	9 (7.0)	0 (0)	0 (0)	3 (2.3)	0 (0)	0 (0)	15 (11.6)

The immunohistochemical scores in PTCs, nodular hyperplasia, and normal thyroid tissue specimens were determined as the multiplication of proportion score and intensity score.

**Table 3:** Correlation of HIF-2*α*, TWIST, and CXCR4 protein expression with clinicopathological parameters in 129 PTCs.

Characteristics	Case (*n*)	HIF-2*α*			TWIST			CXCR4		
		Low	High	*P* value	Low	High	*P* value	Low	High	*P* value
Tissue type										
Normal thyroid tissue	118	118	0		118	0		118	0	
Nodular hyperplasia	61	61	0	—	61	0	—	61	0	—
PTC	129	65	64	<0.001^a^ <0.001^b^	73	56	<0.001	58	71	<0.001^a^ <0.001^b^
Classic PTC	80	40	40	0.980	44	36	0.963	39	41	0.672
Follicular variant of PTC	21	11	10		12	9		9	12	
Tall cell variant of PTC	15	7	8		9	6		5	10	
Oncocytic variant of PTC	13	7	6		8	5		5	8	
Age (years)										
≤45	69	35	34	0.935	43	26	0.159	33	36	0.483
	60	30	30		30	30		25	35	
Gender										
Male	32	16	16	0.960	20	12	0.437	13	19	0.570
Female	97	49	48		53	44		45	52	
Tumor size (cm)										
≤2.3	83	45	38	0.243	51	32	0.135	40	43	0.322
	46	20	26		22	24		18	28	
TNM stage										
I-II	69	35	34	0.935	41	28	0.487	31	38	0.993
III-IV	60	30	30		32	28		27	33	
Lymph node metastasis										
Absent	61	46	15	<0.001	50	11	<0.001	40	21	<0.001
Present	68	19	49		23	45		18	50	

*P* values derived using chi-square test to compare the expression of HIF-2*α*, TWIST, and CXCR4 between subgroups defined by each clinicopathological parameter; ^a^significant difference between PTCs and normal thyroid tissues; ^b^significant difference between PTCs and nodular hyperplasia. *P* < 0.05 significant difference.

**Table 4:** Correlation of HIF-2*α*, TWIST, and CXCR4 protein expression with one another in 129 PTCs.

Proteins	HIF-2*α*				CXCR4			
	Low	High	*r*_*s*_	*P* value	Low	High	*r*_*s*_	*P* value
TWIST								
Low	49	24	0.382	<0.001	46	27	0.414	<0.001
High	16	40			12	44		
CXCR4								
Low	42	16	0.398	<0.001				
High	23	48						

*P* values for Spearman rank test; HIF-2*α*, TWIST, and CXCR4 were tested pairwise; *P* < 0.05 significant difference.

**Table 5:** Correlation of concomitant expression of HIF-2*α*, TWIST, and CXCR4 with LNM.

		LNM	
	Absent	Present	*P*-value
HIF-2*α*/TWIST			<0.001^a^
(1) Both HIF-2*α*/TWIST low expression	43 (87.8)	6 (12.2)	
(2) One of HIF-2*α*/TWIST high expression	13 (32.5)	27 (67.5)	0.032^b^
(3) Both HIF-2*α*/TWIST high expression	5 (12.5)	35 (87.5)	
TWIST/CXCR4			<0.001^a^
(1) Both TWIST/CXCR4 low expression	41 (89.1)	5 (10.9)	
(2) One of TWIST/CXCR4 high expression	14 (35.9)	25 (64.1)	0.018^b^
(3) Both TWIST/CXCR4 high expression	6 (13.6)	38 (86.4)	
HIF-2*α*/CXCR4			<0.001^a^
(1) Both HIF-2*α*/CXCR4 low expression	38 (90.5)	4 (9.5)	
(2) One of HIF-2*α*/CXCR4 high expression	18 (46.2)	21 (53.8)	<0.001^b^
(3) Both HIF-2*α*/CXCR4 high expression	5 (10.4)	43 (89.6)	
HIF-2*α*/TWIST/CXCR4			
Not all of HIF-2*α*/TWIST/CXCR4 high expression	58 (62.4)	35 (37.6)	<0.001^c^
All of HIF-2*α*/TWIST/CXCR4 high expression	3 (8.3)	33 (91.7)	

Correlation of concomitant expression of HIF-2*α*, TWIST, and CXCR4 with LNM were measured by chi-square test; ^a^significant difference among the three groups; ^b^significant difference between group (2) and group (3); ^c^significant difference between groups with and without concomitant expression of all three molecules.

**Table 6:** mRNA expression of HIF-2*α*, TWIST, and CXCR4 in PTCs, nodular hyperplasia, and normal thyroid tissues.

Tissue	Case (*n*)	HIF-2*α*		TWIST		CXCR4	
		ΔCT, mean ± SD	*P* value	ΔCT, mean ± SD	*P* value	ΔCT, mean ± SD	*P* value
Normal thyroid tissue	10	2.9 ± 0.6	—	2.8 ± 0.7	—	4.4 ± 1.3	—
Nodular hyperplasia	10	3.1 ± 1.3	0.940	3.4 ± 1.0	0.111	5.1 ± 1.6	0.226
PTC without LNM	15	3.7 ± 1.3	0.101	21.0 ± 9.1	<0.001^a^	28.4 ± 8.6	<0.001^a^
PTC with LNM	15	4.3 ± 1.7	0.467	28.3 ± 9.5	0.005^b^	37.8 ± 7.4	0.010^b^

Mean ± SD of HIF-2*α*, TWIST and CXCR4 mRNA expression in normal thyroid and nodular hyperplasia tissues, PTCs without LNM and PTCs with LNM after normalization to GAPDH (Mann-Whitney *U* test, ^a^significant difference between PTCs without LNM and normal thyroid tissues; ^b^significant difference in HIF-2*α*, TWIST, and CXCR4 mRNA expression between PTCs with LNM and PTCs without LNM).
